# Surgery

**Published:** 1837-04

**Authors:** 


### SURGERY.

### *On the Treatment of Vesico-Vaginal Fistulæ.* By Professor DIEFFENBACH.

AFTER having, in the manner of most writers on this disease, entered into a consideration of the difficulties which attend any attempt at its radical cure, M. Dieffenbach describes the operation which he usually performs in these cases. Before the operation, the rectum is evacuated. The patient is then placed in the same position as for lithotomy, the back being flat, and the head placed upon a small pillow. Five or six assistants are necessary. Dieffenbach first injects the bladder with cold water. When the fistulous opening is small, the escape of the water indicates its situation, and, when the edges of the fistula are cut, washes away the blood from them. The divided speculum is introduced, and then the vagina is caught hold of in the vicinity of the fistulous opening, with a pair of hooked forceps (hakenzange). The speculum being now withdrawn, the vagina is pulled downwards, the border of the fistula seized with a pair of simple forceps, perforated with a knife, and considerable strips of it are cut away. When the opening is very large, its vaginal borders are cut away some lines distant from its vesical border, in order to make a large wounded surface. This is not possible in small fistulæ. In the latter case, Dieffenbach removes a triangular portion, the middle of which is the fistulous opening; the apex of the triangle is directed towards the bladder; and thus he forms a large wounded surface. The sutures, consisting of strong and numerous threads, are applied whilst the vagina is drawn out, by means of small curved stitching needles. If the parts are hard and unyielding, the sutures are best introduced, according to the structure and seat of the fistula, by straight needles, three-quarters of an inch in length, or by small and very strongly curved needles, introduced by means of a needle-holder. The threads are easily tied by the fingers, and may either be cut short off, or, being allowed to hang from the vagina, may be attached by adhesive plaster to the labia. The patient is then put to bed, an elastic catheter is allowed to remain in the bladder, and an œsophagus tube in the vagina. Through each tube, cold water is injected every half-hour, which escapes again. The water is used less as an antiphlogistic, than as a diluent of the urine: diminishing its injurious action upon the wound in the bladder, and cleansing the mucus from the vagina. Both tubes should lead into a urinal. After four, five, or six days, the sutures are taken away. The speculum of Kluge is the best instrument to employ, and the threads should be seized with forceps and cut with scissors. If any suture has cut through, or if the whole operation has failed, the borders of the wound may be smeared with tincture of cantharides; but if, as most probably will be the case, this is not followed by success, the operation must be repeated. The general treatment in strong and young people, must be the most antiphlogistic. Should an acute inflammation of the bladder occur, it is generally fatal; but this may be easily prevented. Dieffenbach treats the patient, after this operation, as he does cases of penetrating wounds of the abdomen or thorax. If cystitis occur, leeches should be abundantly applied to the vagina, after which the bleeding is very considerable, if lukewarm water is injected; and the pains in the bladder frequently cease in a short time. The only food allowed is mucilaginous drink; the only medicine, an emulsion of castor oil with cherry-laurel water. If the stools become very frequent, the decoction of althæa with cherry-laurel water, or the emulsion of almonds with the same water, are administered.

*Medicinische Zeitung*, No. 36. 1836.

---

*On Pessaries, and the Radical Cure of Prolapsus Vaginæ et Uteri.*
By Professor Dieffenbach.

This distinguished surgeon has long discontinued the use of pessaries in his own practice. To them he ascribes the occurrence of many diseases of the vagina and uterus, as well as of the neighbouring parts; and although he admits that there may be cases in which their use is likely to be beneficial, he considers that such cases are comparatively very rare. He was led to adopt the mode of practice which he here recommends, by seeing the case of a woman, the subject of prolapsus of the vagina and uterus, in whom parts of the vagina sloughed, during its state of prolapse: the uterus and vagina were replaced whilst granulation was going on, and the result was a complete cure of the disease. The first case with which Dieffenbach met, after this, on which he was determined to imitate the natural process, was that of a woman with prolapsus of the uterus, which could be easily replaced, but as easily prolapsed, when it was not kept in by a sponge.

The operation was thus performed. The bladder and rectum were emptied; the uterus was made to prolapse, and a portion of about the size and shape of a hen's egg was removed from the left side of the vagina, the sharper end of which was directed backwards, the opposite end forwards, and came in contact with the nymphæ. The fold was then seized with a pair of forceps, the uterus being previously pressed somewhat backwards to take off the tension of the vagina, and then dissected out with a slightly curved scalpel. The same process was repeated on the right side. The wound was cleansed, and at its hinder part two sutures were applied, the uterus was next replaced, and three other sutures were applied within the vagina. Had all the sutures been completed before the attempt was made to replace the uterus, it is possible that its reduction could not have been effected. Some little irritation followed, which ceased, however, on the removal of two of the sutures from either side. On the sixth day, all the sutures had separated.

Since the time at which Dieffenbach performed this operation, he has repeated it very often. He now employs a smaller number of sutures; usually only two, and never more than three. In many cases he uses no sutures at all, as the borders of the wound in the vagina mostly lie close in contact after the uterus has been replaced. The suture is required where there is great relaxation, and a want of irritability of the vaginal membrane; on the other hand, when the individual is robust and the vagina thick, it is better to dispense with sutures. When the surface of the vagina is mortified, it is necessary to fill it with charpie. Tepid mucilaginous injections should be used for some days, and after these, cold water. If, when cicatrization is going on, there is no evident narrowing of the vagina, a compress of charpie smeared with a resinous ointment, and the repeated application of the lapis infernalis, should be employed.

Dieffenbach has often removed the fold from the vagina after having replaced the uterus, by drawing a portion of the former outwards, and cutting it off by a knife with a sawing motion. This is a far easier mode of operating, but great care is necessary not to injure the bladder or rectum, which may happen if the fold of vagina, when tightly stretched by the forceps, should be cut off too near its base. Sutures are not employed in this case.

The position of the patient in the operation above described, should be the same as that for lithotomy. The state and relations of the rectum and bladder with the vagina and uterus should be ascertained, previous to the operation; of the former, by means of the finger, of the latter, by Desault's silver catheter. The catheter sometimes draws off a quantity of retained urine; the evacuation of the bladder being often rendered very difficult by the prolapse of the uterus.

*Medicinische Zeitung*, No. 31. 1836.

---

*Treatment of some Forms of acute Ophthalmia by the Application of successive Blisters upon the Cutaneous Surface of the Eyelids.* By A. VELPEAU.

THE sudden disappearance, on the occurrence of erysipelas of the face, of some forms of ophthalmia, which had long resisted the usual means of treatment, first led M. Velpeau to the use of blisters in ophthalmia, applied as near as possible to the inflamed part, and therefore upon the eyelids. The advantages resulting from their use, were found to be very considerable, and to be most evident, in those cases where the inflamed vessels were not the same as those the action of which was increased by the use of the blisters; thus, e. g. inflammation of the cornea, the vessels of which are derived from the ciliary branches of the ophthalmic artery, is more benefited by blisters than inflammation of the internal surface of the eyelids, which are supplied by the palpebral branches, and which are directly acted upon by the new cause of irritation. M. Velpeau therefore thinks that blisters applied upon the eyelids will be of the most service in those cases where the inflammation is nourished by the muscular branches of the ophthalmic artery, the ciliary and central of the retina. M. Velpeau has now employed blisters in these cases more than fifty times. In no case have they increased the evil which they were intended to remedy, they have not increased the pain, they leave no indelible marks upon the face, and the only evil effect which has been observed to follow them, is an occasional stye.

The immediate advantages following blisters in such cases, are: diminution of headach, if it previously existed; diminution of lachrymation and intolerance of light, of redness and thickness of the ocular conjunctiva; cleansing of ulcers; lessening of the cloudiness and suffusion of the cornea and aqueous tumor, of effusions of pus or lymph, or at least a discontinuance of their formation, together with improvement in the general state of the patient.

But in many cases, there is a class of secondary effects, which do not clearly manifest themselves before the blistered surface begins to heal. Of these, the most remarkable is the diminution of the cloudiness of the transparent parts of the eye. If lymph be deposited at the bottom of an ulcer; in the substance of the cornea; under the form of hypopium; in layers or masses, it is equally under the power of the blister, disappears as it were by enchantment; so that the clarification of the cornea and aqueous tumour appears to be the special object of the blister. Another effect, almost as constant but not so rapid as the preceding, is the extinction of inflammation in the conjunctiva, then in the cornea. If there is chemosis, this gradually diminishes. Should ulcers be formed on the cornea, when the inflammation is calmed, there will require other remedies to hasten their cicatrization. Blisters applied in front of the orbit are not beneficial in all forms of ophthalmia. They are of especial advantage in favouring the absorption of matters which tend to obscure the clearness of the transparent media of the eye; and their use is indicated in acute inflammations, foreign to the eyelids; in inflammations of the various tunics of the eye, and of the parts contained within the orbits. In ophthalmies which are seated in the fibro-serous tissue of the eye, i. e. in rheumatic ophthalmies, the effects of blisters are more complete than in any other cases, whatever may be the intensity of the inflammation. No topical application is so efficacious. The disease is, as it were, extinguished beneath the blister, and ordinarily disappears entirely by the use of one or two blisters in the space of from eight to fifteen days. The catarrho-rheumatic ophthalmia is still more under the influence of the blisters, applied upon the eyelids. M. Velpeau concludes his paper by hinting at the possible advantage to be derived from blisters in the earliest period of cataract; he grounds the idea of their possible utility, on the influence which they appear to possess of restoring those parts of the eye which have become cloudy, to their natural transparency; but the notion is supported by no facts.

*Journal des Connaissances Médico-Chirurgicales. Septembre,* 1836.

---

*Treatment of Club-Foot with Plaster of Paris.* By Professor Dieffenbach.

Dr. Dieffenbach has employed the following treatment of club-foot, during the last six years, and its advantages are farther commended by the success of other surgeons who have employed it. The apparatus of which he makes use, consists of an oaken box, the sides and ends of which may be taken off: internally it is very smooth. After having oiled the inside of the box, together with the leg and foot, the latter are held within the box in their normal position, whilst the gypsum properly prepared is poured into it; the limb, as it were, swimming in the preparation. As the gypsum begins to harden, the hand must be gradually withdrawn; still, however, holding the extremity of the foot until it is quite consolidated. The toes and metatarsus project beyond the gypsum.

This treatment has been borne very well during a continuance of three or four weeks, and in one case only was it necessary to discontinue it, from the pain which it occasioned. The loss of power which the healthy limb suffers during the necessary confinement, Dr. Dieffenbach considers as an advantage, as both extremities thus acquire a more nearly corresponding strength. The application may be renewed by taking away with a chisel the upper surface of the gypsum; when the limb may be lifted out, washed, oiled, and replaced; the gypsum which was cut away, being substituted by another portion.

After a month's continuance of this treatment, and when the limb has acquired a better position, the following application is substituted. Broad strips of adhesive plaster are so applied as to keep the limb in a proper position, and the foot and leg are carefully bandaged with a roller. A very diluted mixture of gypsum is then spread over the bandage with a large brush, and this having dried, the same is repeated three or four times. On the following day the whole is covered with a varnish, consisting of rosin and spirit of wine (one drachm of the former to one ounce of the latter). Such a bandage is capable of being worn for months without occasioning the least inconvenience. But should pain be complained of from any cause, e. g. an excoriation, a portion of the dressing may be cut away at the part, and when the sore is healed, a reapplication of adhesive plaster and gypsum to the part is all that is necessary.

If after a time the limb acquires its entirely normal form, in children who are not very young, the following application is employed, which allows them to walk about, and effects a complete cure on another principle. The apparatus consists of (1), a sole made of felt; 2, a thin wooden splint, one inch and a half in breadth, and reaching from the knee to the sole of the foot; 3, strips of adhesive plaster; 4, a roller of the same breadth, and five yards in length; 5, a solution of rosin in spirit. The felt-sole, spread with sticking-plaster, is applied to the foot. A strip of plaster is then carried from the back of the foot, over its inner border, obliquely across the sole, thence outwards and upwards in front of the ankle, to terminate spirally in the upper part of the calf. Two other strips are similarly applied; the one before, the other behind the first. The whole is then enveloped by some spiral turns of the bandage. A long piece of adhesive plaster, one inch and a half in breadth, is folded so as to adhere, except in the middle, where it forms a noose. It is then applied to the inner side of the leg and sole, to which it adheres, whilst the noose extends to the outer border of the foot. Into this noose is inserted the lower end of the splint, in which two notches are made to receive the plaster. The splint is then pressed and fastened to the whole limb by more strips of adhesive plaster. From the foot to the knee a roller is now to be most carefully applied, to be sewn together at each turn, and finally to be covered with the solution of rosin.

The peculiarity of this dressing, is, not simply to maintain the limb in an unchanged position, but to bend the foot in an opposite direction. For as the individual places his foot upon the ground, the splint which projects one-third or one-half of an inch beyond it, first touches the ground, the foot is thus somewhat bent outwards. Walking in the room is therefore of advantage, as it remarkably contributes to restoration. If the patient goes out, he should wear a broad soft laced boot, in the thin sole of which an aperture should be left, through which the splint may project. *Wochenschrift für die Gesammte Heilkunde.* No. 27. 1836.

---

*On the Permanent Immoveable Bandage.* By DR. SUETIN.

DR. SUETIN has employed for some time, a bandage constructed upon the same principles as those which Larrey and subsequently Dieffenbach recommended in certain cases of fracture, deformity, &c.: but he has introduced a modification which, from considerable experience, he is disposed to regard as an improvement; and he conceives that its application will be attended with benefit, in a much greater number of cases, than those in which it has hitherto been employed.

The chief advantages attending the use of the permanent immoveable bandage, and which contrast it with those commonly employed, are, that when applied to a limb, it fits itself to all the inequalities of its surface, constitutes a compact covering, no one part of which can he moved without the simultaneous motion of the whole. Its action is not upon particular parts of the limb, but upon its whole circumference, and it is thus a splint in every direction. When applied, it fulfils a threefold indication: it maintains permanent coaptation; opposes constantly the action of forces which tend to disturb the relations of the reduced fracture; and effects all this, by a uniform, regular, and methodic compression. These are advantages rarely to be obtained by the ordinary means, and they are such as render the bandage a valuable acquisition in many other cases, than in those of fractured limbs. And even in cases of compound fracture, where great suppuration is to be anticipated, the application of this bandage has appeared very much to limit the process of suppuration. In this observation Dr. Suetin agrees perfectly with Larrey.

Inflamed articulations, where rest is desired; diseased joints, where the object is to effect anchylosis in a favorable position; club-foot and analogous deformities of the limbs, and other obvious cases, indicate the application of this permanent bandage.

The material employed by Dr. Suetin, recommends itself by its cheapness, and by the facility with which it may at all times be obtained. The essential parts of the apparatus are a roller or many-tailed bandage, pasteboard splints, and a solution of starch in water of a thick mucilaginous consistence.

The general mode of its application, varying in some degree according to the parts to which it is applied, is as follows: Enclose the limb in the roller or bandage, and then smear its whole surface with the solution of starch. Over this again pass another roller and let an assistant spread the starch over each turn as it is made. When this has been done, apply the pasteboard splints, which must be cut and moistened so as accurately to correspond to the limb. Dr. Suetin recommends that the pasteboard should be torn instead of cut, as it has not then a sharp and irritating edge. When the pasteboard is also smeared with starch, pass the last bandage over the whole. Of course, in the application of this treatment to fractures of different kinds, the particular circumstances of such fractures, and the general principles of treatment will require certain modifications, which it is needless to specify.

*Bulletin Médical Belge,* No. 11. *Novembre,* 1836.

---

*Local Treatment of certain Forms of Venereal Disease, followed in the Berlin Charité Hospital.* By Dr. Strunz.

*Orchitis.* The compression of the testicle by strips of adhesive plaster, of a quality as little irritating as possible, generally constitutes the whole of the treatment of hernia humoralis occurring in connexion with gonorrhœa or gleet. The surprisingly favorable operation of this mode of treatment, has been shown in all the cases to which it has been applied. In general, let the inflammation be of a high or low degree, the testicle is enveloped in plaster so soon as the patient is placed under treatment, and the application is usually well borne, even when it causes at first an increase of pain. This pain ceases soon after the testicle is enveloped, and when the patient lies in bed with the organ maintained by a small cushion in an elevated position, he will be quite at ease in a few hours, even when the inflammation is most active. As the testicle diminishes in size, and the plaster becomes loose, it will require to be renewed, and to be repeated until the disease is quite terminated. The advantage of this treatment does not consist simply in the rapid and easy stop which is thus put to the inflammation itself, but in the prevention of that induration which is so often among its most irremediable effects. In the rarest forms of orchitis, where the inflammation is very intense, and is connected with extreme sensibility, the use of compressing plasters is preceded for a day or two by emollient poultices.

*Venereal Warts.* M. Strunz states that it is useless to attempt to eradicate these warts, until they have finished their growth and have become *ripe.* The sign of their having attained this state, consists in a whitish or greyish colour of the points of the warts, such as they would present if lightly touched with blue stone. If it is endeavoured to destroy them before they have attained this state, the effort will almost certainly be fruitless; as they almost always reappear. At the period of their ripening, M. Strunz believes it to be immaterial what means are employed for their destruction, whether cutting or caustic; and he recommends nothing new. He regards as of great importance the observation of Fricke, that small excrescences are often so concealed in the mucous crypts at the entrance of the vagina, as to be overlooked in a cursory examination: and that thus, females are regarded as healthy who are not so. The perfect cure of these must be effected by completely destroying the little crypts, by means of some potential cautery.

*Medicinische Zeitung,* Nos. 33, 34. 1836.

*Cure, after Excision of a Portion of Intestine.* By Professor DIEFFENBACH.

A STRONG man, aged fifty, had suffered for fourteen days from strangulated inguinal hernia of the right side. Several ineffectual attempts at replacement had been made. At this time, Dieffenbach saw the patient. In addition to the usual symptoms, there was reason to suspect sloughing of the protruded parts, and escape of fæcal matters into the hernial sac. An incision of about three inches in length was made into the swelling; when there escaped an ichorous fluid, with fæcal matter, and portions of mortified intestine. The diseased intestine was drawn outwards, and three inches of it, which were partially mortified, softened and thickened, together with a corresponding portion of mesentery, were cut away. A small artery of the mesentery required to be tied, and the ligature was cut close to the knot. During this process, the ends of the intestine were held by assistants. The angular incision in the mesentery was first united by ligature; and then the extremities of the divided intestine, by means of separate threads, so inserted as to bring the peritoneal coats alone into connexion. The mucous membrane was not perforated. The parts were then carefully replaced. Shortly afterwards, castor oil was administered, and repeated with some croton oil, until very large evacuations were produced. These were followed by great improvement in all the symptoms. Mild aperients and the antiphlogistic regimen were the only means required during the process of cure, which was complete in the fourth week after the operation.

The individual returned to his usual employment, which was laborious, and some weeks subsequently, after very hard work and the use of very indigestible food, he was suddenly seized with all the symptoms of intussusception, with which he died. Two diseased conditions were found within the abdomen. In the left lumbar region, a portion of small intestine had coiled around, strangulated and become adherent to, another portion of the same gut; above this, the ileum and jejunum were much inflamed, adherent and covered with flakes of lymph, and contained a large quantity of excrementitious fluid, which was also found in the duodenum and stomach. The ileum, particularly near the strangulated part, was very much distended by this fluid. Beneath the strangulation, the intestine was empty and contracted, passing in this state to the right inguinal aperture, at which many convolutions were closely adherent. Whilst dividing the false membrane which united the intestine to the inner parietes of the abdomen at this part, a drop of pus was found surrounding a ligature. Here was the part of intestine which had been operated on. It was closely adherent to the abdominal parietes and the contiguous convolutions of intestine. On cutting it open, the extremities which had been joined together by ligatures, were found to be connected by a smooth cicatrix, interrupted only by the situations of the ligatures, still suppurating. The ligatures were adherent, and their extremities lay in the cavity of the intestine. The portion of intestine beyond, about a span in length, terminated in the cæcum. Nothing worthy of notice was found in the other organs.

*Wochenscrift für die gesammte Heilkunde.* No. 26. 1836.

---

*Report of the Cases treated at the Ophthalmic Hospital of the Imperial High-School at Vienna, during the Session* 1833-4. By Professor ROSAS.

THE number of patients treated in this institution during the academical year 1833-4 was 1,132; of which, 120 were in-patients, and 1012 out-patients. Of the former, 72 were males, 48 females; 4 were under 10 years of age, 29 under 20, 35 under 30, 19 under 40, 6 under 50, 10 under 60, 13 under 70, 4 under 80. With respect to diseases, the numbers were as follows:—Phlegmonoid inflammation of the eyelids, 1; Erysipelatous ditto, 1; Inflammation of the meibomian glands, 1; Lagophthalmos, 1; Cancer of the eyelid, 1; Various forms of conjunctivitis, 25; Chronic ophthalmo-blenorrhœa, 2; Trachoma (granular conjunctiva), 2; pterygium, 1; Encanthis, 1; Ulceration of the angle of the eye, 1; Fistula lachrymalis, 1; Inflammation of the globe of the eye from various causes, 20; Amaurotic weakness of sight, 3; Gutta serena, 3; Opacities of the cornea, 3; Ulceration of the cornea, 5;

Cicatrix of the cornea with closure of the pupil, 1; Adhesion of the iris to the cornea (synechia anterior), 3; Hydrops oculi, 1; Effusion of blood into the tissues of the eye, 1; Prolapsus of the iris, 2; Contraction of the pupil from effused lymph, 1; Cataract, 29; Staphyloma, 7; Medullary fungus of the eye, 1; Melanosis of ditto, 2.

The cases of phlegmonoid and erysipelatous inflammation of the eyelids did not present any thing worthy of notice. The same thing may be observed of the cases of inflammation of the ciliary glands and lagophthalmos.

The cancer of the eyelids occurred in a locksmith, aged thirty-three, who had repeatedly suffered from affections of the eyes. In his thirtieth year, he had a violent inflammation of the eyelids, terminating in a tumour of the external angle of the left eye, which gradually increased in size and hardness, assumed a dark red colour, became extremely painful, and finally exhibited the characters of a cancerous ulcer. At the period of his admission, it was three-quarters of an inch in length, and an inch in breadth. An issue was made in the left arm, and kept open, an appropriate diet prescribed, and the patient was ordered to take the extract. conii, in doses which were gradually increased to the amount of half a drachm in the day. The ulcer was covered with pulvis Cosmii (Frere Cômes' paste); and, when the slough dropped off, the surface was dressed with Hellmund's salve, to which a little pulv. cosmii was added. This application was continued until the hard cancerous base of the ulcer was destroyed, and then it was dressed with a pledget dipped in Sydenham's liquid laudanum. In a few weeks the cicatrization was complete, and there was no return of the disease.

With respect to the cases of conjunctivitis, the furuncular form occurred in 2; the catarrhal in 7, (in 4 as lippitudo, in 2 as blepharo-blenorrhœa, in 1 as ophthalmo-blenorrhœa); the catarrho-rheumatic in 7; the scrofulous in 8; and the gonorrhœal in 1. The treatment of these cases was conducted in the usual way, and proved completely successful, except in two cases of catarrhal inflammation in which some derangement of vision remained, and in a third case where the sight had been wholly impaired by an opacity of the cornea. The case of gonorrhœal ophthalmia also terminated unfavorably. The usual antiphlogistic and alterative means were promptly applied, and the discharge from the urethra, which had become suppressed on the occurrence of the conjunctivitis, was restored by inoculating with matter taken from the diseased eye; but the symptoms had gone too far, and the case terminated in suppuration of the cornea.

The cases of trachoma were of catarrho-rheumatic origin, affected both eyes, and had been of several months' standing, when the patients were admitted. They were treated with leeches, scarifications, the lapis divinus of Beer, white precipitate, and other alterative and astringent remedies, with considerable benefit; but the patients left the hospital before the cure was complete.

The case of fistula lachrymalis arose from a neglected lippitudo. The duct was dilated by means of small catgut bougies, and the patient was obliged to wear a hollow lead style for several months.

Among the cases of inflammation of the globe of the eye were observed,—of rheumatic ophthalmia, 12, (5 as scleritis, 1 as keratitis, 3 as internal ophthalmia, 2 as general acute ophthalmia, and 1 as chronic ophthalmia); scrofulous ophthalmia, 3, (1 as keratitis, 2 as internal ophthalmia); syphilitic iritis, 1; traumatic ophthalmia, 1; panophthalmitis, 3. One of the cases of rheumatic ophthalmia terminated in opacity of the retina and mydriasis. The case of traumatic ophthalmia, and one of the cases of panophthalmitis, terminated in atrophy of the globe of the eye. The rest were cured or relieved.

One of the cases of amaurotic weakness of sight occurred in a peasant girl of plethoric habit, who laboured under amenorrhœa. The pupil was small, the iris sluggish, vision myopic and indistinct. Bleeding from the feet, leeches to the mammæ and perineum, cold affusion, warm pediluvia, and the use of aloetic medicines, restored the menstrual secretion, and removed every trace of amaurosis. In the second case, the disease was of a rheumatic and congestive nature. The pupil was natural, the iris sluggish, the patient complained of pain in the eye and orbit, intolerance of light, and obscure vision. He was treated with cream of tartar, and afterwards with small doses of tartar emetic and blisters to the nucha. A saturated solution of extract. hyoscyami (gr. j. ad gr. viij. aq. distill.) was dropped into the eye daily. He recovered completely. The third case was of long standing, and resisted every form of treatment.

One of the cases of gutta serena occurred in a cabinet-maker, aged thirty. The right eye, which was first attacked, was quite blind; the left still retained some sensibility to light. The patient had been subjected to intermittent fever, and since his twenty-fifth year, had been frequently annoyed with a defluxion from the nostrils and wandering rheumatic pains. The motions of the eye were unsteady, the cornea less convex than usual, the iris sluggish, the pupil small, and forming a vertical oblong. He was directed to take the tartar emetic mixture, and use the decoct. althæ, with manna, as a sternutatory. In ten days he could discern large objects with the left eye, and could distinguish the light with the right. The vomiting, however, produced by the tartar emetic was so annoying to him, that he would not submit to this mode of cure any longer, and left the hospital. The second case was one of complete amaurosis of both eyes. The patient was a Jew of weak habit, who laboured under great irritability of the nervous system, with involuntary emission of semen, and was supposed to be addicted to masturbation. Various remedies were tried for the space of six months, but without any effect. The third case was also treated without success. The patient had imperfect amaurosis of the right, and perfect amaurosis of the left eye, with adherent cataract. The iris was pale and sluggish, and the pupil contracted in both eyes; in the left, the crystalline lens was opaque, and adhered to the iris.

In a case of partial chalk-like opacity of the cornea of the right eye, and purulent opacity of the cornea of the left, with adhesion to the iris, *iridodialysis* was performed in the first, and diaresis in the latter case, with the most favorable results. In a case of partial chalk-like opacity of the cornea, caused by organized lymph, in which the power of vision was limited to mere sensibility to light, the operation for *iridectome-dialysis* was performed without success; the new pupil closed again. In a third case, with opacity from organized lymph, and a varicose state of the ciliary bodies of both eyes, in a scrofulous girl, aged eighteen, the operation was equally unsuccessful.

Of the five cases of ulceration of the cornea, three were the result of traumatic keratitis, one of rheumatic inflammation of the cornea, and one of small-pox. As long as any irritation remained, a few drops of the saturated solution of extract of hyosciamus were dropped into the eye once or twice daily, and a weak collyrium of corrosive sublimate with tincture of opium, was subsequently employed.

In a case of complete opacity of the cornea after small-pox, with atrophy of the left eye, *kerectomia* was performed on the right eye, which was still sensible to light, but without advantage.

The same operation was performed, and with a similar result, in a case of adhesion of the iris to the cornea, arising from ophthalmo-blenorrhœa. A case of contraction of the pupil from adhesion of the iris to the cornea, was successfully treated by performing irido-dialysis, after Langenbeck's method. The result was equally favorable in a similar case treated in the same way.

The case of hydrops oculi, which arose from ophthalmia of a rheumatic and scrofulous character, was considerably improved by repeatedly puncturing the cornea, and the use of derivatives and other appropriate means.

The case of effusion of blood into the tissues of the eye, which was produced by a blow of a hammer, yielded completely to vigorous antiphlogistic treatment.

The two cases of prolapsus of the iris arose from perforating ulcers of the cornea. By the use of proper antiphlogistic means, and the constant application of the saturated solution of extract of hyosciamus, they were both cured, leaving behind only a slight anterior synechia, which caused very little disturbance of vision.

In a case of contraction of the pupil from bands of lymph, with subacute iritis, mercurialization, derivatives, and the use of the saturated solution of extract of hyosciamus, produced the most remarkable improvement; the patient, who at the period of his admission could merely distinguish day from night, was capable of discerning very small objects when he left the hospital.

Of 29 individuals who laboured under cataract, 14 were males, 15 females. Of this number, 1 was under 10, 3 under 20, 1 under 30, 1 under 40, 7 under 60, 12 under 70, and 4 under 80 years. With respect to the origin of the disease, 2 were congenital, 4 traumatic, 5 from latent internal ophthalmia, 1 from habits of intoxication, 2 from abdominal derangement, 1 from gout, 1 from excessive weeping, 13 from old age or early decrepitude. Thirty-four cataracts were operated on; 13 by extraction, all or most of which were hard cataracts. Discission through the cornea was performed in two cases of soft capsular cataract in grown persons; discission through the sclerotic in 8 cases, (chiefly in young subjects,) of which 4 were membranous, 2 hard, and 2 soft. Couching through the cornea was performed in 3 cases, 1 complicated with gout, 2 with abdominal derangement. Couching through the sclerotic in 8 cases, most of which were hard, lenticular, or membranous cataracts. In all cases, where any complication existed likely to exercise any influence on the results of the operation, the utmost care was taken to palliate or remove it before operation. The accidents consequent on the different modes of operation were, after extraction, two cases of mild acute, and one of chronic iritis; after discission through the cornea, a slight kerato-iritis; after discission through the sclerotic, a mild sclero-choroideitis; after depression through the cornea, a slight keratitis. The results of extraction were favorable in twelve cases. Discission through cornea produced good vision in two cases, and through the sclerotic in eight cases. Depression through the sclerotic produced the same favorable result; in one case of depression through the cornea, the result was marred by a chronic iritis, in the other two it was successful.

The staphyloma was partial in two cases, and complete in six. The usual operation was performed in all with favorable consequences. A case of incipient medullary sarcoma was treated with calomel, the external use of the Naples ointment, and topical bleedings, and terminated in atrophy of the eye. A melanotic degeneration of the right eye, to which warm fomentations of cicuta, mallows, and hyosciamus were applied as a preliminary measure, with the view of relieving pain, gradually yielded, and terminated in atrophy of the eyeball. In another case of the same kind, extirpation of the eye was performed with success. After the operation, the patient used solvent and alterative remedies, and wore an issue in the left arm.

The number of external patients treated at this institution was 1012; of which, 567 were males, and 445 females. Of these were treated, in October, 87; November, 69; December, 52; January, 72; February, 72; March, 92; April, 123; May, 149; June, 116; July, 83; August, 45; September, 52. In 282, the right eye was affected; in 280, the left; in 450, both eyes.

*Medicinische Jahrbücher des Öst. St.,* xx. Band, i. Stuck. 1836.

---

*Treatment of Artificial Anus.* By M. BLANDIN.

AN artificial anus, in this case, was the consequence of a strangulated inguinal hernia, in which six inches of intestine had become gangrenous. The two extremities of the intestine were parallel to one another; the fæcal matters escaping from the superior extremity, which was very tumid externally. The finger could be readily introduced into it. The inferior extremity was more contracted, and its diameter daily diminished. Having tried various means to reduce the tumor of the upper extremity of the intestine, M. Blandin comprehended it in a ligature, and it separated as a slough on the fourth day afterwards. He then constructed an enterotome formed of two branches, each of which terminated by an oval blade, from eighteen to twenty lines in length, and from six to eight lines in breadth; the internal surface being so undulated that each elevation corresponded to a depression on the opposite blade. The two blades were then introduced, one into the upper, the other into the lower extremity of the intestine; several incisions having been first made upon the circumference of the latter. The blades were inserted to a depth of four or five inches, and compressed by means of a screw. Abstinence and rest were enjoined. No evil consequences ensued. The enterotome separated on the fifth day; its two blades being covered by the two extremities of the intestine, the gangrene of which it had caused. On the same evening, the patient passed solid fæces, per anum, for the first time during an entire month. Gas and a yellowish green fluid alone escaped from the external fistula; and notwithstanding the fact that the patient over indulged in food, the cure was complete in two months after the employment of the instrument.

*Archives Générales de Médecine*, tome xii. *Novembre*, 1836.

---

*Treatment of Chronic Catarrh of the Bladder, by Injections.*
By D. M. DEVERGIE, Sen.

DR. DEVERGIE has recorded eight cases of chronic catarrh, some of long standing, which were cured by injecting balsam of copaiba, into the bladder. Some of these cases had succeeded to an acute cystitis: in others the disease had gradually manifested itself and maintained throughout its chronic character. If stricture of the urethra exist, this requires to be remedied before employing injections. A moderate quantity of an emollient fluid must first be injected, to ascertain the capacity of the bladder, but not in sufficient quantity to irritate it. General means must be resorted to, to calm the inflammation and local pain, the general erethism, &c. Narcotics must next be added to the emollient injections; and these may be repeated three or four times daily. When the state of irritation of the bladder and neighbouring parts is allayed, the copaiba should be injected. A dose of uniform strength is not suited to every case. A drachm of balsam of copaiba to an ounce of barley-water is strong enough to commence with; the quantity of balsam may be increased according to its effects. The combination of narcotics with copaiba renders the latter less exciting. The balsamic injections may be allowed to remain in the bladder for a period of from ten to twenty minutes. The quantity of copaiba is to be gradually augmented; and it should not be injected more frequently than once daily, nor intermitted more than two days. The injection is to be continued until the muco-purulent secretion has quite ceased. It is necessary to guard against the occurrence of inflammation of the mucous membrane of the alimentary canal, and under such a circumstance to suspend the use of the balsamic injections.

*Gazette Médicale de Paris. Octobre*, 1836.

---

*Dissection of a Sclerotic Staphyloma.* By Dr. JAEGER, of Erlangen.

A MAN, aged forty-eight, had for several years been amaurotic in one eye; the cornea was dull, and the upper eyelid depressed. On examining the eye after death, the following was observed:—On the upper segment of the bulb, three lines from the edge of the cornea, was a sclerotic staphyloma, of a globular form, depressed in the middle by the rectus superior muscle, so as to be divided into two portions. It measured one inch long, half an inch broad, and the height of both prominences was two and a half lines. The dark blue colour of the swelling was marked with white striæ and points. Held against the light, it lost its blue colour, and became transparent like the cornea. Dr. Jäger pronounced the tumour to be a collection of water between the cornea and choroid, in consequence of which the sclerotica had become thinned and of a bluish colour. On laying open the eye, the affected part of the sclerotica was found to be quite thin and transparent, and presented only here and there anything of its usual fibrous character. A quantity of limpid fluid was present between the sclerotica and the very thin choroid. The retina was thinner than natural. The vitreous body was small, but not otherwise abnormal.

*Ammon's Zeitschrift für die Ophthalmologie*, V. Bandes, 2 und 3 Heft. 1836.

---

### *Case of Ligature of the Common Carotid.* By Dr. Bedor, Senior Surgeon of the Hôtel-Dieu, of Troyes.

[It would be difficult to find a more exquisite example of the overlaying of a few important facts by a multiplicity of trifling details and an infinity of words, than this case affords. We shall give in a few lines what the author has contrived to spread over twelve large quarto columns.]

A man, aged twenty, was stabbed in a fray, with a sort of workman's punch, small, and shaped somewhat like a foil. He received two wounds, one on the breast and another just below the ear, in front of the mastoid process; and this last, the only one of consequence, had evidently divided some large branch of the external carotid artery, as was manifest from the great and repeated hæmorrhage, which nothing could repress, although attempts to do so were persevered in for sixteen days. The operation was performed in the usual manner, great care being taken not to denude the vessel of its cellular envelope to a greater extent than was necessary to tie as low down as possible, and that nothing was included in the single ligature but the artery itself. Immediately after the operation, great exhaustion and a state of stupor supervened, followed by extreme paleness, almost imperceptible pulse, and slight delirium. He, however, had a good night, and, although the local wounds and the general symptoms were troublesome and somewhat threatening, the intellectual functions being occasionally, but slightly, disturbed, the progress to recovery was regular. The ligature shifted, showing separation, on the eighth day after the operation, but did not come away entirely until the fourteenth. A month after this date, he left the hospital perfectly well.

*La Presse Medicale. Paris, Fevrier* 4, 1337.

